# Increasing averaging beats improves the test accuracy on Holter‐based late potentials in patients with myocardial infarction

**DOI:** 10.1111/anec.13089

**Published:** 2023-09-19

**Authors:** Kenichi Hashimoto, Naomi Harada, Motohiro Kimata, Yusuke Kawamura, Naoya Fujita, Akinori Sekizawa, Yosuke Ono, Yasuhiro Obuchi, Tadateru Takayama, Yuji Kasamaki, Yuji Tanaka

**Affiliations:** ^1^ Department of General Medicine National Defense Medical College Tokorozawa Japan; ^2^ Department of Integrative Physiology and Bio‐Nano Medicine National Defense Medical College Tokorozawa Japan; ^3^ Department of General Medicine Nihon University School of Medicine Tokyo Japan; ^4^ Department of General Medicine Kanazawa Medical College Himi Municipal Hospital Himi Japan

**Keywords:** increase averaging beats of late potential, late potentials, myocardial infarction, signal averaged electrocardiogram, sudden cardiac death, ventricular tachycardia

## Abstract

**Background:**

The prevalence of Holter‐based late potentials (H‐LPs) in cases of fatal cardiac events has increased. Although the noise level of H‐LP is higher than that of conventional real‐time late potential (LP) recording, a procedure to reduce the noise severity in H‐LP by increasing the averaging beats has not been investigated.

**Methods:**

We enrolled 104 patients with post‐myocardial infarction (MI) and 86 control participants. Among the patients, 30 reported sustained ventricular tachycardia (VT), and the remaining 74 had unrecorded VT. H‐LPs were measured twice in all groups to evaluate the efficacy of increasing the averaging beats for H‐LPs. Thereafter, the average of LP was calculated at 250 (default setting), 300, 400, 500, 600, 700, and 800 beats.

**Results:**

Across all three groups (MI‐VT group, MI non‐VT group, and control group), the noise levels significantly decreased in consonance with the increase in averaging beats. In the MI‐VT group, the H‐LP positive rate considerably increased with the increase in the averaging beats from 250 to 800 both at night and daytime. In the MI‐VT group, the LP parameters significantly deteriorated, which led to a positive judgment corresponding to the increment of the averaged night and day beats. The H‐LP positive rates were unchanged in the MI non‐VT and control groups, while the LP parameters remained consistent, despite the increased averaging beats in the MI non‐VT and control groups.

**Conclusion:**

Increasing the calculated averaging beats in H‐LPs can improve the sensitivity of predicting fatal cardiac events in patients with MI.

## Graphical abstract

During daytime, the positive rate of LP significantly increased by 800 averaging beats (*p* = .001. vs. 250 beats averaging). During day‐time, the highest (AUC = 0.845) for averaging beats 800 in daytime. AUC, area under the curve; LP, late potentials; MI‐VT group, post‐myocardial infarction ventricular tachycardia group; MI non‐VT group, post‐myocardial infarction non‐ventricular tachycardia group.

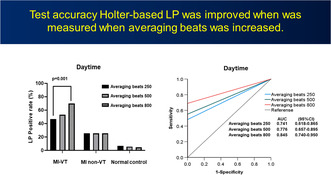

## 1 INTRODUCTION

Ventricular late potentials (LPs), identified based on standard real‐time signal‐averaged electrocardiograms, are a promising predictive tool for prognosticating lethal arrhythmia or sudden cardiac death in patients with various structural heart diseases. These diseases include post‐myocardial infarction (MI) (Gomes et al., 1987; Ikeda et al., 2000), dilated cardiomyopathy (Mancini et al., 1993), arrhythmogenic right ventricular cardiomyopathy (Kamath et al., 2011), and cardiac sarcoidosis (Yodogawa et al., 2018). Recently, Holter‐based LPs (H‐LPs) have been detected using a high‐resolution ambulatory electrocardiogram (HECG) system via commercial means (Hashimoto et al., 2018). Subsequently, the utility of H‐LPs in the prediction of serious cardiac events has been reported in patients with MI (Amino et al., 2019; Gatzoulis et al., 2019). LPs in combination with noninvasive ECG markers such as T‐wave alternans (TWAs), heart rate turbulence (HRT), heart rate variability (HRV), and non‐sustained ventricular tachycardia detected using a high‐resolution Holter ECG system facilitate risk stratification of fatal arrhythmic events and cardiac death with high accuracy (Gatzoulis et al., 2019; Hashimoto et al., 2021; Kinoshita et al., 2020). The advantage of H‐LP is that it can be measured with other noninvasive risk stratification markers, such as TWAs, HRV, and HRT, simultaneously with routine Holter ECG measurements. This is beneficial in saving time for patients and healthcare providers because it shortens procedural duration.

However, H‐LPs possess certain limitations. The noise level of the H‐LP is higher than that of conventional real‐time LP recording. Moreover, during the daytime, H‐LPs are likely to be affected by noise in daily life. High noise levels have been known to mask low‐amplitude LPs, increasing disease underestimation and false negatives and a decrease in sensitivity (Frances, 2010; Steinberg & Bigger, 1989). A noise level under 0.4 μV is desirable for accurate test results (Breithardt et al., 1991); however, most current reports on H‐LPs have been performed at a noise level of 0.8 μV (Amino et al., 2019; Hashimoto et al., 2021; Kinoshita et al., 2020; Yoshioka et al., 2013). Contrastingly, while theoretically increasing the calculated averaging beats leads to a lower noise level, Frances (2010) and Steinberg and Bigger (1989) revealed that the sensitivity of real‐time LP for serious cardiac events such as ventricular tachycardia (VT) in patients of MI improved when noise levels decreased from 1.0 to 0.3 μV by increasing the calculated averaged beats. However, whether an increase in calculated averaged beats can decrease the noise level in the case of H‐LP has not yet been evaluated.

The primary objectives of this study are to (1) reduce the noise level of H‐LPs by increasing the calculated averaged beats in patients with post‐MI and (2) evaluate whether the test accuracy of H‐LPs can be improved by increasing averaging beats.

## 2 METHODS

### 2.1 Study design and ethics

We retrospectively enrolled patients with MI who underwent high‐resolution Holter electrocardiography (HECG) for arrhythmia during their follow‐up after MI between March 2012 and December 2021. Although this was a retrospective study, we enrolled patients who underwent the same sequential order of events and tests: HECG was performed after the MI event, followed by the observation of VT events after HECG recordings. The time interval from the MI event to the date of HECG recordings was 477.0 [69.5, 835.3] (median [interquartile range]) days. The time interval from the HECG recordings to the date of the sVT event was 208.7 ± 360.4 days. We also included 86 participants who were referred to our institute due to complaints of chest symptoms, such as chest pain and palpitation, with no history of a diagnosed cardiac disease in the normal control group. All normal control participants demonstrated sinus rhythm without atrioventricular block or intraventricular conduction delay on 12‐lead ECG. All patients had normal serum electrolyte levels and renal and liver functions. Moreover, none of the patients had a history of cardiac disease or arrhythmia, and none of them reported receiving medications. HECG of the normal control group was performed within 1 month from the first visit to our institution. Among 104 patients with MI, 30 with clinically sustained VT by March 2021 were designated as the “MI‐VT group,” and 74 without sustained VT or ventricular fibrillation as the “MI non‐VT group.” The characteristics of the enrolled patients' post‐MI and the normal control group are described in Table 1. Demographically, there was no age difference between the MI‐VT and MI non‐VT groups, but there was a higher percentage of males in the MI‐VT group (*p* = .008). A coronary artery lesion of the left anterior descending branch was more frequently observed in the MI‐VT group than in the MI non‐VT group (*p* < .001). Moreover, the left ventricular ejection fraction was significantly lower (*p* < .001), and the left ventricular dimension diameter was considerably greater (*p* = .001) in the MI‐VT group than in the MI non‐VT group. Patients presenting coronary artery stenotic lesions in more than two branches were categorized into the MI‐VT group (47%) and the MI‐non‐VT group (52%) (Table 1). Subsequently, PCI was pursued whenever possible during the chronic phase, or alternatively, coronary artery bypass grafting (CABG) was performed. Ongoing patient monitoring was overseen by outpatient physicians who utilized coronary CT and myocardial scintigraphy methods. In the event of restenosis, patients were subjected to PCI. Consequently, by the time the Holter ECG was conducted, residual stenosis was, in principle, absent.

**TABLE 1:** Baseline characteristics of the study patients.

Demographics	MI‐VT group (*n* = 30)	MI non‐VT group (*n* = 74)	*p* Value	Normal control group (*n* = 86)
Age (years)	68.9 ± 11.7	68.0 ± 11.5	.887	56.8 ± 19.7
Sex: male, *n* (%)	29 (97)	62 (84)	.008	0 (0)
Hypertension, *n* (%)	26 (87)	64 (85)	.019	0 (0)
Dyslipidemia, *n* (%)	18 (60)	40 (54)	<.01	0 (0)
Diabetes mellitus, *n* (%)	14 (47)	32 (44)	<.02	0 (0)
Coronary culprit lesion				
RCA	6 (20)	26 (35)	.557	–
LAD	22 (73)	39 (53)	<.001	–
Cx	2 (7)	10 (12)	.103	–
Single vessel disease by CAG	16 (53)	35 (48)	.577	–
Two vessel disease by CAG	8 (27)	21 (28)	.860	–
Three vessel disease by CAG	6 (20)	18 (24)	.635	–
Left main tract disease by CAG	0 (0)	2 (3)	.504	–
CABG	5 (17)	15 (20)	.568	–
Echocardiographic data				
LVEF (%)	46.5 ± 17.2	58.5 ± 12.3	<.001	71.2 ± 5.9
LVDd (mm)	58.4 ± 11.4	50.3 ± 7.3	.001	44.3 ± 4.0
Creatine kinase (U/L)	619 [335, 6031]	488 [265, 1027]	.032	–
Troponin I (ng/mL)	14.7 [1.9, 25.7]	11.4 [4.2, 23.2]	.041	–
NSVT	12 (40)	16 (21)	<.001	
Paroxysmal atrial fibrillation	5 (17)	9 (12)	.051	–
Renal function				
Estimated GFR (mL/min/1.73 m^2^)	52.4 ± 24.9	52.8 ± 24.9	.916	78.0 ± 18.2
NYHA functional class				
NYHA I (%)	14 (47)	58 (79)	<.001	–
NYHA II (%)	9 (30)	15 (20)	<.001	–
NYHA III (%)	5 (17)	1 (1)	.167	–
NYHA IV (%)	2 (6)	0 (0)	–	–
Therapy				
β‐blocker (%)	25 (83)	50 (68)	<.001	–
RAS‐inhibitor (%)	22 (73)	39 (52)	<.001	–
CCB (%)	17 (57)	25 (34)	<.001	–
Diuretic (%)	20 (67)	23 (31)	<.001	–
Amiodarone (%)	10 (33)	4 (5)	<.001	–
Mexiletine (%)	0 (0)	6 (8)	.03	–
Pilsicainide (%)	0 (0)	1 (1.4)	.405	–

*Note*: Data are presented as *n* (%) or mean standard deviation.

Abbreviations: CABG, coronary artery bypass grafting; CAG, coronary artery angiography; CCB, calcium channel blockers; Cx, circumflex branch; GFR, glomerular filtration rate; LAD, left anterior descending; LVDd, left ventricular dimension diameter; LVEF, left ventricular ejection fraction; NSVT, non‐sustained ventricular tachycardia; NYHA, New York Heart Association; post‐MI‐VT, post‐myocardial infarction ventricular tachycardia; RAS, renin–angiotensin system; RCA, right coronary artery.

Sustained ventricular arrhythmias were defined as follows: VT, ≥30 s consecutive ventricular complexes at a rate of >100 bpm (Kinoshita et al., 2020). The presence of NSVT was defined as more than three consecutive ventricular premature beats (VPBs) of more than 100 beats/min, as previously reported (Lin et al., 2016). HECG was performed at least 1 month after the onset of MI. The exclusion criteria were as follows: (1) persistent atrial fibrillation or flutter, (2) right or left bundle branch block and intraventricular conduction delay, (3) permanent pacing, (4) atrioventricular block II–III degree, (5) cases wherein all ECG markers could not be simultaneously measured, and (6) noise level over 1.5 μV at 250 averaging beats. When the noise level exceeds 1.5 μV, the terminal portion of the waveform of the LP is unclear, which poses a challenge when attempting to distinguish the true waveform of LPs from noise.”

### 2.2 Measurement of Holter‐based LPs

LPs were recorded for all patients with MI and normal control participants using an HECG system (Spider View; Ela). ECG data were obtained at a sampling rate of 1000 Hz and 16‐bit A/D conversion. First, the LP‐measurable 24‐h Holter ECG data were recorded for 24 h; then, 20 min, approximately around 0:00 and 12:00 when the noise level is low, was selected as the time frame for the measurement. More specifically, the ECG cutter and dedicated software Syne Scope® (SORIN GROUP) was used to cut out 20 min where the noise level was visually low. The LP parameters (fQRS, LAS_40_, and RMS_40_) were measured and manually edited by a cardiologist or electrocardiography technician. In this frame, all beats of the QRS were checked to confirm that the morphology of the QRS had not changed due to body position or movement. In our study, LPs were averaging 250 beats starting from the first beat. If the noise level exceeded 1.5 μV at that point, the time frame was deemed ineligible, and the investigator attempted to identify another time frame closer to 0:00 and 12:00. In the Spiderview® model used in this study, a template waveform is selected at the beginning of the measurement and only the backward waveforms whose morphology matches the template waveform are averaged by automatic measurement. Thus, the achievement rate was defined as the percentage of the target waveform that is counted when the backward waveform matches the template waveform. For example, if the target number of averaging beats is 800 and the waveform counted and added had averaging beats of 750, the achievement rate was 93.75% (750/800). For LP measurement, ECG data were filtered through a 4‐pole (24 dB/octave) bidirectional filter (finite impulse response [FIR]) with the bandpass ranging from 40 to 250 Hz. Then, the averaging of LP was performed at 250 (default setting), 300, 400, 500, 600, 700, and 800 beats. Orthogonal X, Y, and Z bipolar leads (the number of leads was three) with silver‐silver chloride electrodes (Blue SENSOR®; METS) were used for all H‐LP recordings. Three LP parameters of the H‐LPs were evaluated; these parameters included fQRS and duration of LAS_40_ and RMS_40_ in MI patients and normal control participants. LPs were considered positive when any two of the following criteria were met: fQRS > 114 ms, RMS_40_ < 20 μV, and LAS_40_ > 38 ms (Breithardt et al., 1991).

### 2.3 Statistical analyses

Data are presented as mean ± standard deviation for normally distributed continuous variables and as medians (interquartile range: 25th–75th percentile) for non‐normally distributed variables. Patient characteristics were compared using the *χ* ^2^ test for categorical variables, Student's *t*‐test for continuous and parametric data, and the Wilcoxon rank sum test for nonparametric data. Cochran's *Q* test was performed to compare the LP positive rate among 250, 500, and 800 averaging beats. A receiver operating characteristics (ROC) curve was generated, and the area under the curve (AUC) was calculated to compare the test accuracy for predicting VT among averaging beats 250, 500, and 800. Analysis of variance or Friedman's analysis of variance on rank was used to compare LP parameter values (fQRS, LAS_40_, and RMS_40_) and noise levels between 250 and 800 averaging beats. All statistical analyses were performed using SPSS version 28 software (IBM Corp.); *t*‐tests were two‐sided, and *p* values <.05 were considered statistically significant.

## 3 RESULTS

### 3.1 Signal averaging achievement rate and noise reduction

The average of LPs was sequentially performed from 250 to 800 beats. The noise level notably decreased according to the increment of averaging beats from 250 to 800 beats in the MI‐VT, MI non‐VT, and normal control groups. The results of the averaging beats are described in Table S1. Although the achievement rates were 100% from 250 to 300 beats averaging in the MI‐VT, MI non‐VT, and normal control groups both in daytime and night‐time, the achievement rates ranging from averaging 400 to 800 beats were 92%–99% (Table S1).

### 3.2 Positive rate of LPs

**FIGURE 1:**
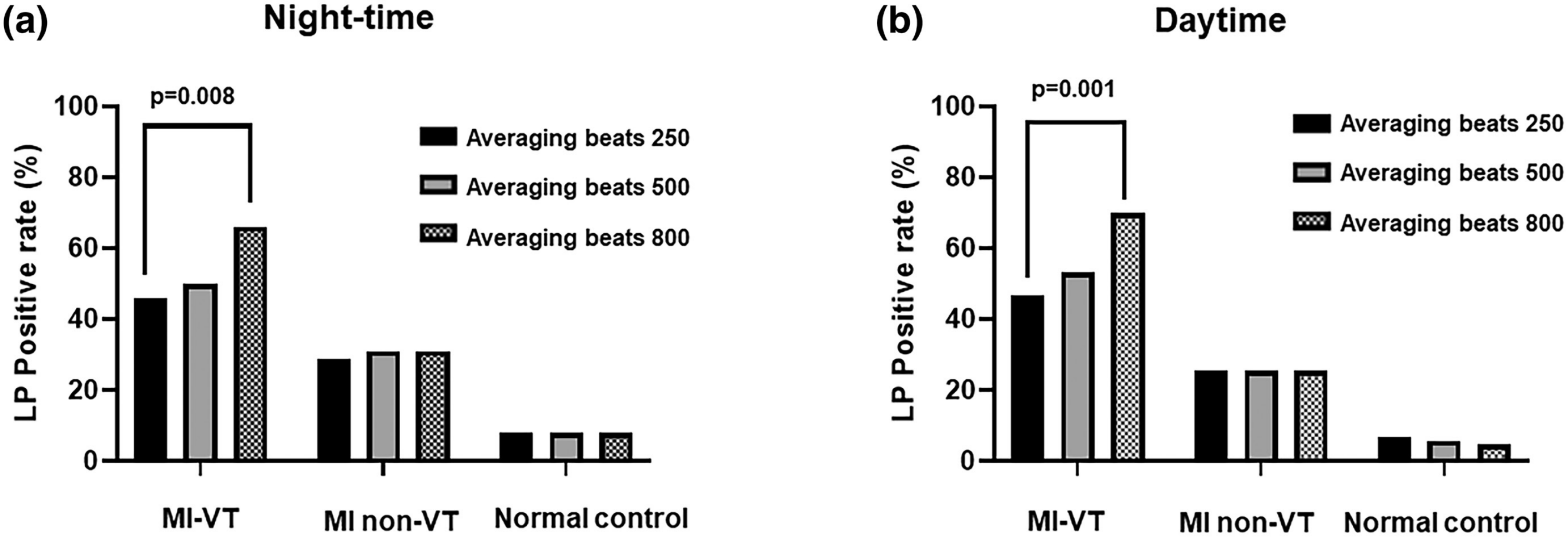
In the MI‐VT group, the positive rate of LP significantly increased at 800 averaging beats (*p* = .008 vs. 250 averaging beats) at night‐time. However, there were no significant changes in the LP positive rate in the MI non‐VT and healthy groups at night‐time (a). During daytime, the positive rate of LP significantly increased by 800 averaging beats (*p* = .001 vs. 250 beats averaging). No significant changes were observed in the LP positive rate in the MI non‐VT and normal control groups at night‐time (b). LP, late potentials; MI‐VT group, post‐myocardial infarction ventricular tachycardia group; MI non‐VT group, post‐myocardial infarction non‐ventricular tachycardia group.

**FIGURE 2:**
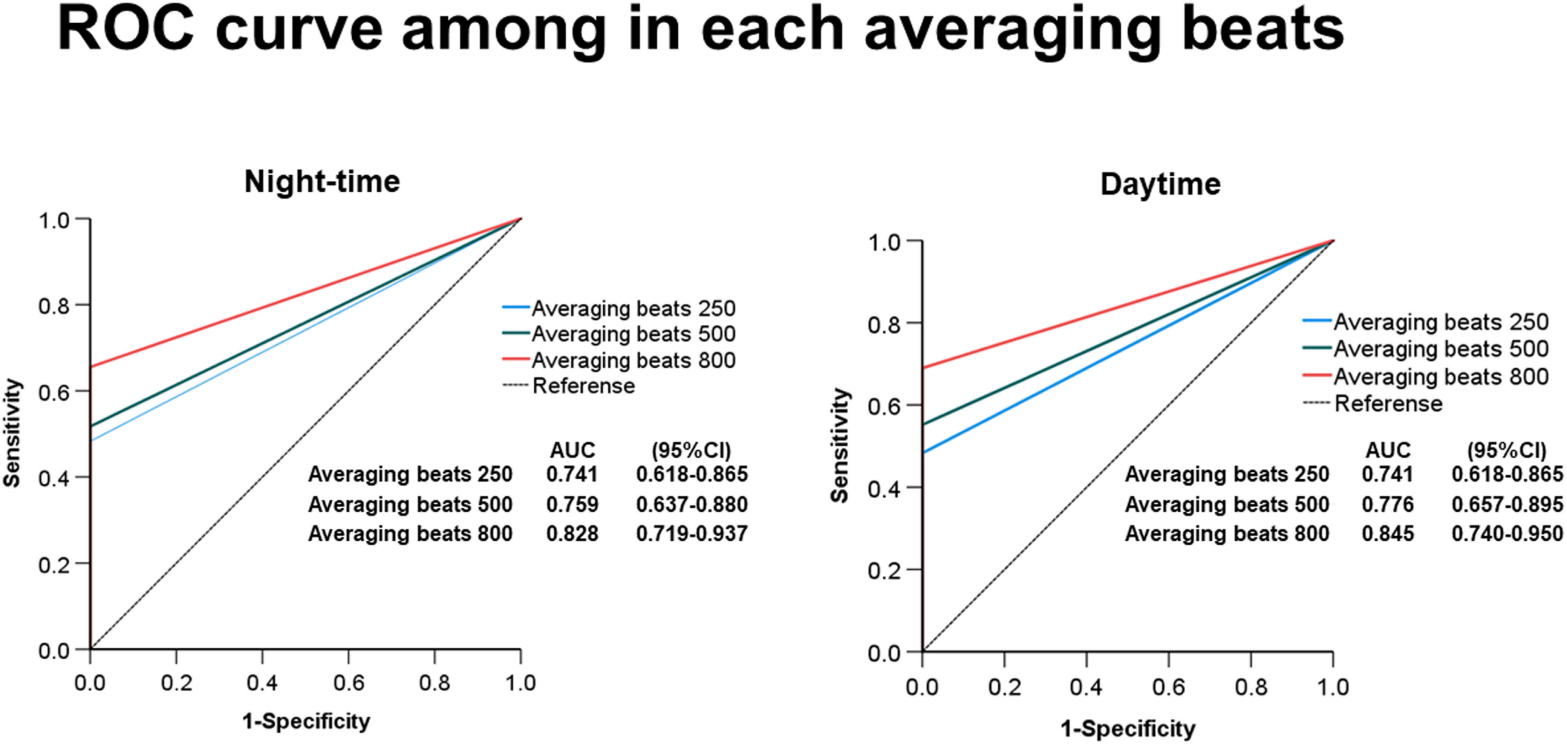
ROC curves for the averaging beats (250, 500, and 800 beats). At night‐time, the AUC was the highest (AUC = 0.828) for averaging beats 800 (a), and the highest (AUC = 0.845) for averaging beats 800 in daytime (b).

Figure 1a exhibits the positive rate of LPs in the MI‐VT, MI non‐VT, and normal control groups. At night, the positive rate of H‐LPs significantly increased when the averaging beats was increased to 800 beats (*p* = .008) in the MI‐VT group. In contrast, in the MI non‐VT group, the positive rate of LPs did not change significantly by increasing the averaging time from 250 to 500 or 800 beats. In addition, in the normal control group, the positive rate of LPs did not change significantly when the averaging beats were increased to 500 or 800 beats. During the daytime, as in the night‐time, the LP positive rate increased significantly in the MI‐VT group by increasing the number of averages from 250 to 800 beats (*p* = .001), whereas the LP positive rate remained unchanged in the MI non‐VT and normal control groups even when the number of averages was increased to 500 or 800 beats. There was no significant change in the LP positive rate among 250, 500, and 800 averaging beats; however, two cases that were initially positive turned negative due to the increasing of averaging beats during the day. In the normal control group, the positive rates with 250, 500, and 800 averaging beats were 9% (8/86), 8% (7/86), and 7% (6/86), respectively (Figure 1b). Table 2 lists the number of participants in all LP patterns in each group. In the normal control group, two cases showed a positive to negative pattern as the averaging increased. Besides these two patients, increasing the averaging beats in the measurement of LP may have resulted in a positive LP but not in a negative LP. In the ROC curve for each averaging beats in night, the AUC was the highest (AUC = 0.828) for averaging beats 800 (Figure 2a). Similarly, the AUC was the highest (AUC = 0.845) for averaging beats 800 in the daytime as well (Figure 2b).

**TABLE 2:** Patient numbers and patterns of LP determination in each category.

Averaging beats	MI‐VT group (*n* = 30)		MI non‐VT group (*n* = 74)		Normal control group (*n* = 86)	
250 → 500 → 800	Night‐time	Daytime	Night‐time	Daytime	Night‐time	Daytime
Number of “negative → negative → negative”	11	10	51	55	80	80
Number of “negative → negative → positive”	4	4	0	0	0	0
Number of “negative → positive → positive”	1	2	1	0	0	0
Number of “positive → positive → positive”	14	14	22	19	6	4
Number of “negative → positive → negative”	0	0	0	0	0	0
Number of “positive → positive → negative”	0	0	0	0	0	1
Number of “positive → negative → negative”	0	0	0	0	0	1
Total number	30	30	74	74	86	86

### 3.3 Values of Holter‐based LP

Table 3 lists the values of LP parameters (fQRS, RMS_40,_ and LAS_40_) for individual averaged beats (250, 500, and 800 beats). In the night‐time, the values of fQRS and LAS_40_ were considerably prolonged with the increment in averaging beats (fQRS, *p* < .05 for 250 vs. 500 beats, *p* < .001 for 250 vs. 800 beats; LAS_40_, *p* < .05 for 250 vs. 500 beats, 250 vs. 800 beats in each). Conversely, RMS_40_ significantly decreased according to the increment of averaging beats (*p* < .001 for 250 vs. 800 beats). Briefly, the LP parameters worsened according to the increment of averaging beats. These results support the results of LP positivity rates among the MI‐VT, MI non‐VT, and normal control groups. This trend was also observed during the daytime. Similarly, the LP parameters worsened by increasing the average beats during the day, with no change in the MI non‐VT and normal control groups (Table 4). The detailed value results of fQRS, RMS_40_, and LAS_40_ in signal averaging from 300 to 800 beats, including 250 beats, are shown in Tables S2 and S3. Moreover, the detailed results of the noise level in the MI‐VT, MI non‐VT, and normal control groups in signal averaging from 300 to 800 beats, including 250 beats, are demonstrated in Tables S4 and S5. Increasing the averaging beats from 250 to 800 at night‐time significantly worsened the LP parameter only in the MI‐VT group, while there was no change in the MI non‐VT and normal control groups. This trend was also observed during the daytime (Tables S4 and S5). Table S6 compares the clinical background between the LP determinate change (+) and LP determinate change (−) groups according to the increment of averaging beats. As in Table 1, the groups with and without change in LP were compared in terms of history, coronary culprit lesion, echocardiographic index, NHYA classification, and medications taken; there were no significant differences between the groups. Table 5 shows the mean heart rate during averaging period for the MI‐VT, MI non‐VT, and normal control group groups. No significant differences were observed between the groups or in the number of averages during nighttime measurements. However, during daytime measurements, the MI‐VT group exhibited a tendency towards bradycardia in comparison to the MI non‐VT group. Notably, the MI‐VT group displayed significantly more pronounced bradycardia than the normal control group (*p* < .05).

**TABLE 3:** Values of LP parameters among 250–800 averaging beats (night‐time).

fQRS values			
Averaging beats	250	500	800
MI‐VT group (*n* = 30) (ms)	112.0 [105.5, 138.0]	113.0 [105.5, 138.0]*	117.0 [106.0, 140.0]^#^
MI non‐VT group (*n* = 74) (ms)	102.0 [93.0, 112.5]	103.3 [93.8, 113.3]	102.5 [94.0, 113.3]
Normal control group (*n* = 86) (ms)	91.4 ± 7.5	91.2 ± 7.6	91.2 ± 7.5
RMS_40_ values			
Averaging beats	250	500	800
MI‐VT group (*n* = 30) (μV)	24.0 [12.0, 51.0]	24.0 [12.0, 48.5]	22.0 [12.0, 43.0]^#^
MI non‐VT group (*n* = 74) (μV)	29.5 [18.8, 48.0]	29.0 [17.5, 45.3]	29.0 [17.5, 47.3]
Normal control group (*n* = 86) (μV)	45.0 [28.0, 64.0]	45.0 [27.8, 66.3]	46.0 [27.8, 65.0]
LAS_40_ values			
Averaging beats	250	500	800
MI‐VT group (*n* = 30) (ms)	35.0 [26.0, 47.5]	36.0 [27.5, 47.5]*	38.0 [28.0, 48.0]*
MI non‐VT group (*n* = 74) (ms)	31.0 [23.0,40.3]	31.5 [24.0, 40.3]	31.5 [23.8, 40.3]
Normal control group (*n* = 86) (ms)	29.7 ± 7.2	29.7 ± 7.3	29.6 ± 7.4

Abbreviations: LAS_40_, low‐amplitude signals <40 μV in the terminal filtered QRS complex; LP, late potential; MI, myocardial infarction; fQRS, filtered QRS duration; RMS_40_, root mean square voltage of the terminal 40 ms in the filtered QRS complex; VT, ventricular tachycardia.

*p < .05; ^#^ *p* < .001.

**TABLE 4:** Values of LP parameters among 250–800 averaging beats (daytime).

fQRS values			
Averaging beats	250	500	800
MI‐VT group (*n* = 30) (ms)	116.0 [104.0, 136.5]	117.0 [105.0, 137.5]	117.0 [105.5, 138.0]
MI non‐VT group (*n* = 74) (ms)	98.0 [89.5, 110.0]	98.0 [90.0, 110.5]	98.0 [90.0, 110.5]
Normal control group (*n* = 86) (ms)	86.3 ± 8.1	86.0 ± 7.9	86.1 ± 7.8
RMS_40_ values			
Averaging beats	250	500	800
MI‐VT group (*n* = 30) (μV)	24.0 [11.0, 55.0]	20.0 [11.0, 51.0]	18.0 [12.0, 46.0]^#^
MI non‐VT group (*n* = 74) (μV)	37.0 [22.0, 52.0]	37.0 [21.5, 49.0]	37.0 [21.5, 51.0]
Normal control group (*n* = 86) (μV)	55.0 [33.0, 83.0]	58.5 [33.0, 84.5]	57.0 [33.8, 83.3]
LAS_40_ values			
Averaging beats	250	500	800
MI‐VT group (*n* = 30) (ms)	38.0 [26.5, 48.5]	39.0 [27.0, 48.0]*	39.0 [27.0, 48.0]^#^
MI non‐VT group (*n* = 74) (ms)	29.0 [22.5,34.0]	29.0 [23.0, 34.5]	29.0 [23.0, 34.5]
Normal control group (*n* = 86) (ms)	26.7 ± 7.3	26.8 ± 7.1	26.8 ± 7.1

Abbreviations: LAS_40_, low‐amplitude signals <40 μV in the terminal filtered QRS complex; LP, late potential; MI, myocardial infarction; fQRS, filtered QRS duration; RMS_40_, root mean square voltage of the terminal 40 ms in the filtered QRS complex; VT, ventricular tachycardia.

*p < .05, ^#^ *p* < .001.

**TABLE 5:** Values of HR 250–800 averaging beats.

Night			
Averaging beats	250	500	800
MI‐VT group (*n* = 30)	62.5 [55.8, 75.3]	63.0 [55.0, 75.0]	62.0 [55.5, 75.0]
MI non‐VT group (*n* = 74)	61.0 [56.8, 71.0]	61.0 [56.8, 69.8]	61.0 [56.0, 70.5]
Normal control group (*n* = 86)	62.5 [55.0, 69.3]	62.5 [54.8, 69.0]	62.5 [55.0, 69.0]
Daytime			
Averaging beats	250	500	800
MI‐VT group (*n* = 30)	67.0 [64.5, 74.0]	67.0 [64.0, 74.5]	66.0 [64.0, 74.5]
MI non‐VT group (*n* = 74)	69.0 [61.0, 78.5]	69.0 [60.8, 79.0]	69.5 [61.0, 78.0]
Normal control group (*n* = 86)	70.5 [62.0, 81.3]	73.5 [63.8, 80.0]	75.5 [64.5, 81.5]*

Abbreviations: MI, myocardial infarction; VT, ventricular tachycardia.

* *p* < .05 (vs. MI‐VT group, Averaging time 800).

### 3.4 Cases

**FIGURE 3:**
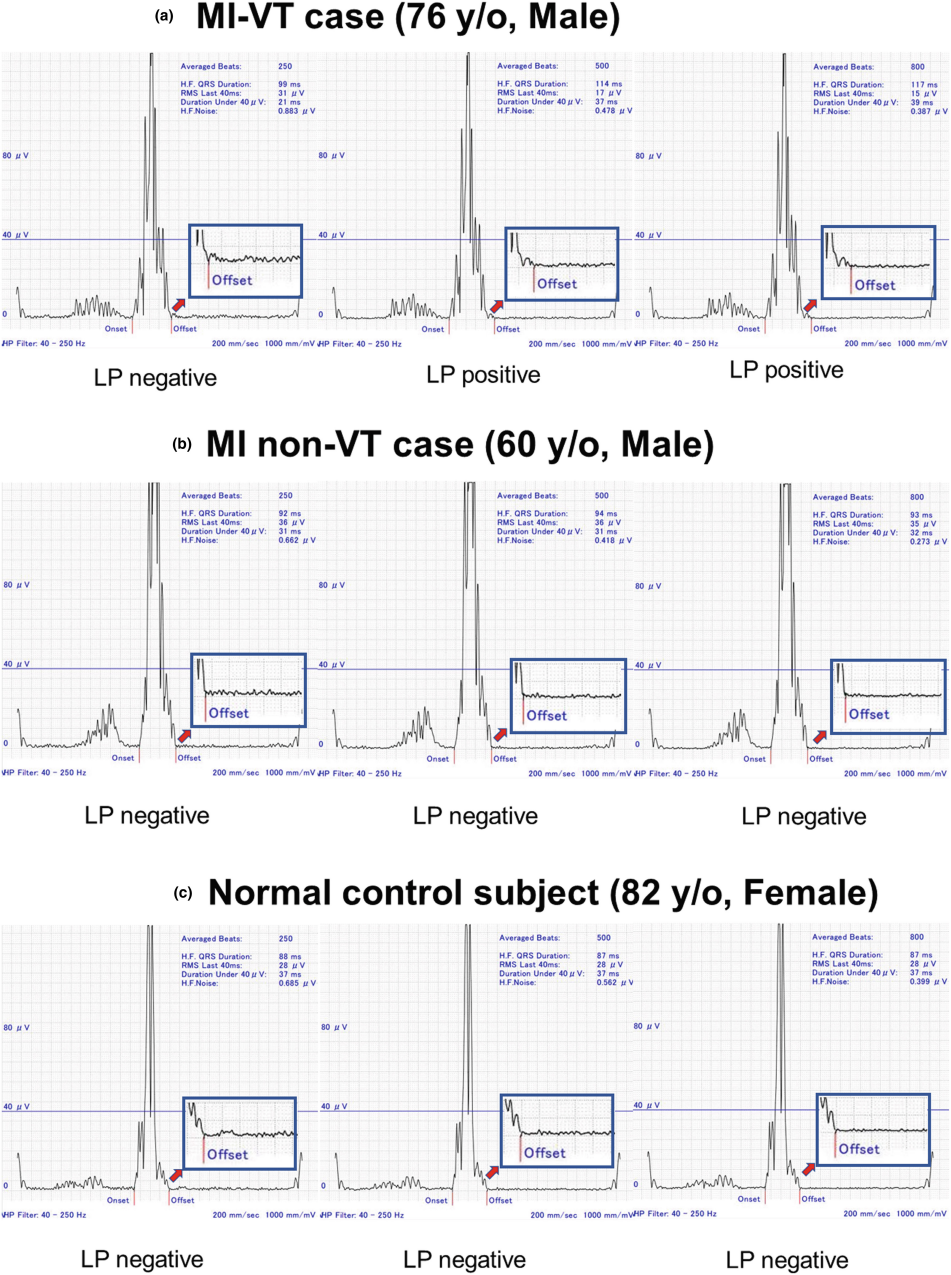
Three representative cases of MI‐VT, MI non‐VT, and normal control. In total, 250 beats averaging of MI‐VT resulted in a high base noise level (0.883 μV) and a negative LP result. Subsequent 500 and 800 beats averaging of MI‐VT resulted in reduction of the noise level (0.478 and 0.387 μV for MI non‐VT and normal control participants, respectively). After averaging 500 and 800 beats, the unclear off set (terminal portion) of LP by the noise level became substantial. The LP was positive (a). Conversely, in the MI non‐VT case and healthy case, the LP did not become apparent even if the noise of the base was decreased by averaging 500 and 800 beats, and the LP result was negative in all settings (averaging 250, 500, and 800 beats) (b, c). MI non‐VT group, post‐myocardial infarction non‐ventricular tachycardia group; MI‐VT group, post‐myocardial infarction ventricular tachycardia group.

Figure 3 shows representative cases of an MI‐VT, an MI non‐VT, and a normal control participant. In the MI‐VT case (a 76‐year‐old male), averaging 250 beats resulted in a high base noise level, while 500 and 800 averaging beats in the MI‐VT case resulted in a reduction in noise. The increment of averaging beats in the MI‐VT group resulted in a change from an unclear LP owing to the noise into a more substantial LP. Contrastingly, in the MI non‐VT participant (a 60‐year‐old‐male) and normal control participant (an 82‐year‐old female), LP did not appear even with the reduction in noise level by signal averaging 500 and 800 beats, after which the test result of LP remained negative.

## 4 DISCUSSION

In patients with MI and normal control participants, increasing the average beats of H‐LP from 250 to 800 beats significantly reduced the noise level both during the night‐time and daytime. Corresponding to the increment of averaging beats, the LP positive rate considerably only increased in the MI‐VT group, with no reported changes in the MI non‐VT and normal control groups. For the MI‐VT group, the LP parameters (fQRS, RMS_40_, and LAS_40_) significantly became worse by the increment of averaging beats, but they remained unchanged in the MI non‐VT and normal control groups. The sensitivity of H‐LP in the MI‐VT group improved as the noise level decreased, whereas the specificity did not change significantly.

The noise component of LP is expressed by the following equation (Steinberg & Lander, 1993):

$$\sum_{J=1}^{R}\mathrm{nj}\left(i\right)/R.$$

The noise level is reduced by a factor of R, when R is the number of beats averaged. Therefore, theoretically, the noise level should decrease as the number of averages increases. It has been reported that noise levels can decrease in real‐time LP measurements when the number of the averaging beats increases (Frances, 2010; Maounis et al., 1997; Steinberg & Bigger, 1989). However, few studies have evaluated changes in LP by increasing the number of averages in H‐LP. The results of the present study are noteworthy in that, not only at night but also during the daytime, when the presence of noise is generally higher, the noise level decreases, and the LP positivity rate becomes significantly higher only in the MI‐VT group. There was no significant change in the positive rate in the MI non‐VT and normal control groups. Therefore, increasing the averaging time improves LP measurement sensitivity. These results may be because the LP parameter values changed around the cut‐off value of the positive criteria with an increase in averaging. For example, the median values of fQRS in the night‐time in the MI‐VT group were 112.0 ms for 250 beats averaging, 113.0 ms for 500 beats averaging, and 117.0 ms for 800 beats averaging. The fQRS exceeded the cut‐off value of 114 ms when the averaging beats were more than 600 beats at night (Table S3). In contrast, the median for the MI non‐VT group was 102–103 ms even after 250–800 cycles, while the median for the normal control group remained in the 91‐ms range.

With respect to the VT‐MI group, it is speculated that very small LPs, which were originally masked by the noise in 250 averaging beats, became detectable after increasing the number of averaging beats to 500 or 800 beats. However, with respect to the MI non‐VT and normal control groups, LPs did not become apparent even if the number of averaging beats was increased as they never existed originally. In addition, when HECG was performed, none of the patients had residual ischemia after clinical testing. However, the possibility of latent ischemia cannot be ruled out completely, given the retrospective nature of this study. It has been reported that LPs do not become positive with transient ischemia induced by exercise stress test or balloon inflation in the coronary artery during coronary artery angiography (Caref et al., 1990; Turitto et al., 1990). Therefore, even in the unlikely event of mild post‐infarction angina, there would be no significant impact on LP. Overall, there were no differences regarding biological rationale between the MI‐VT group and the MI non‐VT or normal control group.

In the MI‐VT and MI non‐VT groups, there were no cases in which the LPs were initially positive and then became negative after 500 or 800 averaging beats (Table 2). However, two patients in the control group were initially positive but became negative after averaging during the daytime examination. The course of one of the two patients was positive (250 beats averaging) → positive (500 beats averaging) → negative (800 beats averaging), and the course of the other patient was positive (250 beats averaging) → negative (500 beats averaging) → negative (800 beats averaging). We speculated that the main reasons for this change in the normal control group are as follows. When the noise level becomes lower at 500 and 800, averaging beats, the low voltage potential, which can also be observed at 250 beats averaging, becomes clear; this low voltage potential is recognized as noise and not an LP. However, it is unclear why this occurs only in the normal control group but not in the MI groups. After reviewing all cases, it was difficult to distinguish MI‐VT cases from control cases with the unaided eye, and there was no obvious difference in the waveforms between the two groups, suggesting that pathological factors were not the cause. For the above reasons, increasing the beats averaging in the control group at a certain frequency will cause some LP positive cases to turn negative (false positives; approximately 1%–2%, or 1–2/86). However, because negative cases are overwhelmingly more common in the control group, the present data suggest that only in the control group LP positive cases are converted to negative cases.

In this study, the MI‐VT group was significantly more likely to use β‐blockers than the MI non‐VT group (Table 1). Thus, although there was no significant difference at night, the MI‐VT group exhibited a tendency towards bradycardia during daytime compared to the MI non‐VT and Control groups (Table 5). Theoretically, the appearance of bradycardia could contribute to an increase in fQRS and LAS_40_, thereby facilitating fulfillment of the positive criteria for diagnosis. The observed dissimilarity in the usage of β‐blockers may have played a role in supporting the outcomes of the present study.

In recent years, the evidence for H‐LP for serious cardiac events has increased (Amino et al., 2019; Gatzoulis et al., 2019; Hashimoto et al., 2021; Turitto et al., 1990). The strength of H‐LPs is that the data of LPs can be collected along with routine analysis in ordinary Holter ECGs. Therefore, they are highly versatile. In recent years, the indices of depolarization abnormalities such as the wavelet transform method (Yodogawa et al., 2011) and fragmented QRS (Luo et al., 2020) have been reported in patients with MI. However, they have not yet been widely used as commercial clinical indices. Therefore, LP, which reflects depolarization abnormalities, remains the gold standard noninvasive ECG marker as a risk stratification index for serious cardiac events, such as lethal arrhythmia or sudden cardiac death. Furthermore, Holter LPs reportedly fluctuate throughout the day (Hashimoto et al., 2021), and the diurnal fluctuation of LP should be considered to further improve LP test accuracy. However, since the total amount of data in 24‐h LP analysis is enormous, the burden on physicians and technicians may increase, which hinders its implementation. Recently, with advances in artificial intelligence (AI) technology, the accuracy of ECG analysis at the μV level has been reported to improve (Shimizu et al., 2022).

In the future, analysis of the Holter LP will be supported by AI. Therefore, the results of this study will be useful for future advances in Holter LP analysis using AI technology. In such a case, the additional increment in averaging beats from 500 to 800, such as 500 and 800 beats, may contribute to improving the accuracy of the H‐LP result.

### 4.1 Clinical implication

The gold standard for the upper threshold of permissible noise levels in late potential (LP) analysis is documented as being below 0.4 μV (Breithardt). However, attaining this precise level in Holter‐based LP assessments is challenging. Therefore, conventionally, a standard of 0.8 μV, twice the 0.4 μV, is acceptable (Amino et al., 2019; Hashimoto et al., 2021; Kinoshita et al., 2020; Yoshioka et al., 2013) clinically. However, since the pros and cons of this have not been thoroughly tested, the noise level is better under 0.4 μV, or even if it exceeds 0.4 μV, it is better as low as possible. Based on the results of this study, it is strongly recommended to average up to 800 times when performing Holter‐based LP in Post MI patients if the noise level is 0.4 μV or higher. Even if the noise level is less than 0.4 μV, averaging up to 800 times can increase sensitivity without increasing the number of false positives. Further studies are desirable, including those on other cardiac diseases.

## 5 LIMITATIONS

This study has several limitations. This HECG system (Spider view) allows signal averaging up to 1000 beats; however, we did not demonstrate averaging over 800 beats. When the averaging number was set to 800, 100% averaging was completed up to 300 beats, but for ≥400 beats, the mean achievement rate of averaging beats was only 92%–99% (Table S1). Moreover, even when 900 or more additional averaging was performed, the average number of signal averages was less than 800. Therefore, we concluded that averaging more than 800 beats was not meaningful. The reasons for this phenomenon are as follows: First, the QRS template waveform initially selected may deviate owing to changes in respiratory status or other biological conditions when the number of signal averaging is higher, resulting in beats that are not averaging. Therefore, it is impossible to perform 100% averaging at higher averaging beats. Second, changes in body position may cause discrepancies between the QRS template waveform and the actual waveform, which is followed by the QRS template waveform in the LP measurements. It has been reported that the LP positive rate generally changes with postural changes such as supine, sitting, or standing position (Yoshioka et al., 2015). The model used in this study does not use an accelerometer. Although coherent body position was confirmed to some extent by the activity record card, it may not be precise, leading to a possibility of changes in body position varying between the patients.

The variable time interval between occurrence of the MI event and the AECG recordings, spanning 477.0 [69.5, 835.3] days (median [interquartile range]), could potentially pose a limitation for retrospective studies such as the present one. This variability underscores the potential challenge of observing consistent electrophysiological characteristics across all participants due to the differing time frames involved. Reentry circuits reflected by LPs are established only 2–4 weeks post‐MI onset, and the positive rate of LPs is variable (Grimm et al., 1988; Ozawa et al., 1987). Therefore, to ensure a uniform timeframe for observation, all patients were enrolled in this study at least 1 month post‐MI onset. However, a possible limitation of the present study is the scarce data verifying LPs post‐MI onset over several years. It has been hypothesized that a new ischemic event has the most significant impact on the electrical substrate. However, all enrolled participants in this study were out‐patients at the institution; from the onset of MI to the HECG procedure, the absence of restenosis was demonstrated in the participants using modalities, such as coronary computed tomography angiography and cardiac scintigraphy, confirming that there were no new ischemic events.

## 6 CONCLUSION

This study demonstrated that the noise level in the MI‐VT group significantly decreased at night and during the daytime by increasing the number of averages. In both night‐time and daytime, the positive rate of H‐LP increased significantly only in the MI‐VT group as the number of increments increased to 500 and 800, while the positive rate did not change in the MI non‐VT group or the normal control group. Therefore, averaging beats more than the default 250 beats in the H‐LP measurement increases the test result's sensitivity. Prospectively, the versatility of LP measurement by Holter ECG is anticipated to expand when AI supports the H‐LP calculation. Consequently, our findings contribute to improving the accuracy of H‐LP as a risk stratification tool for lethal arrhythmia or sudden cardiac death in patients with MI.

## AUTHOR CONTRIBUTIONS

KH, NH: conception and design of the study, data collection, writing of the manuscript, and formatting and submission of the manuscript. YKasamaki: design, conception, and supervision of the study, and revision of the manuscript. MK, YKawamura, NF, AS, YOn, YOb, TT, YKasamaki and YT: supervision of the study, and revision of the manuscript. All authors contributed to the article and approved the submitted version.

## FUNDING INFORMATION

This work was supported by Grants‐in‐Aid for Scientific Research in Japan (JSPS KAKENHI) (Grant Number 20K07816) provided by the Japanese Society for the Promotion of Science.

## CONFLICT OF INTEREST STATEMENT

The authors declare that the research was conducted in the absence of any commercial or financial relationships that could be construed as a potential conflict of interest.

## ETHICS STATEMENT

The study was conducted in accordance with the Declaration of Helsinki and was approved by the Competent Authorities and Ethics Committees of the participating centers (Nihon University School of Medicine and National Defense Medical College, approval no. MF‐2302‐0063 and 4692). Written informed consent was obtained from all patients.

## Supporting information


Tables S1

## Data Availability

The original contributions presented in this study are included in the manuscript/Supplementary material; further inquiries can be directed to the corresponding author.
