# Supplementary material for: Increasing averaging beats improves the test accuracy on Holter‐based late potentials in patients with myocardial infarction
Source: Ann Noninvasive Electrocardiol. 2023 Sep 19;28(6):e13089. doi: 10.1111/anec.13089 (PMC10646378; doi:10.1111/anec.13089)
Supplement: Supplementary file 1 — Tables S1 [file ANEC-28-e13089-s001.docx]

Supplementary Material

Increasing averaging beats improves the test accuracy on Holter-based late potentials in patients with myocardial infarction

**Short title**: Accuracy on H-LPs in MI

Kenichi Hashimoto MD, PhD, ISHNE-F ^1*^, Naomi Harada MA ^1^, Motohiro Kimata MD ^1^, Naoya Fujita MD ^1^, Yusuke Kawamura MD ^1,2^, Akinori Sekizawa MD ^1^, Yosuke Ono MD, PhD ^1^, Yasuhiro Obuchi MD, PhD ^1^, Tadateru Takayama MD, PhD ^3^, Yuji Kasamaki MD, PhD ^4^, Yuji Tanaka MD, PhD ^1^

^1^Department of General Medicine, National Defense Medical College, Tokorozawa,

Saitama, Japan

^2^Department of Integrative Physiology and Bio-Nano Medicine, National Defense Medical College,

Tokorozawa, Japan

^3^Department of General Medicine, Nihon University School of Medicine, Tokyo, Japan.

^4^Department of General Medicine, Kanazawa Medical College Himi Municipal

Hospital, Japan

*** Corresponding author:**

Kenichi Hashimoto

Department of General Medicine, National Defense Medical College, 3-2 Namiki, Tokorozawa City, Saitama 359-8513, Japan

Phone: +81-42-991-1211 (ex. 3633)

Fax: +81-42-995-1238

Email: [hashimoto.kenich2@gmail.com](mailto:hashimoto.kenich2@gmail.com)

| Table S1. | | | | | | | |
| --- | --- | --- | --- | --- | --- | --- | --- |
| **Night-time** | | | | | | | |
| Averaging beats | 250 | 300 | 400 | 500 | 600 | 700 | 800 |
| MI-VT group (mean±SD) (beats) | 250±0 | 300±0 | 400±0 | 500±0 | 600±0 | 697±1 | 784.0±41.0 |
| Mean achievement rate (%) | 100 | 100 | 100 | 100 | 100 | 99 | 98 |
|  |  |  |  |  |  |  |  |
| MI non-VT group (mean±SD) (beats) | 250±0 | 300±0 | 398.7±11.4 | 497.7±24.7 | 589.0±41.6 | 673.4±70.0 | 747.0±102.7 |
| Mean achievement rate (%) | 100 | 100 | 99 | 99 | 98 | 96 | 93 |
|  |  |  |  |  |  |  |  |
| Normal control group (mean±SD) (beats) | 250±0 | 300±0 | 396.0±22.5 | 491.7±40.5 | 585.9±61.4 | 679.3±83.7 | 761.4±107.8 |
| Mean achievement rate (%) | 100 | 100 | 99 | 98 | 98 | 97 | 95 |
|  |  |  |  |  |  |  |  |
|  |  |  |  |  |  |  |  |
|  |  |  |  |  |  |  |  |
| **Daytime** | | | | | | | |
| Averaging beats | 250 | 300 | 400 | 500 | 600 | 700 | 800 |
| MI-VT group (mean±SD) (beats) | 250±0 | 300±0 | 400±0 | 497.9±11.1 | 591.4±33.6 | 682.9±58.9 | 766.4±87.6 |
| Mean achievement rate (%) | 100 | 100 | 100 | 99 | 99 | 97 | 96 |
|  |  |  |  |  |  |  |  |
| MI non-VT group (mean±SD) (beats) | 250±0 | 300±0 | 398.1±16.7 | 495.3±29.4 | 583.9±50.3 | 663.0±81.9 | 733.2±118.0 |
| Mean achievement rate (%) | 100 | 100 | 99 | 99 | 98 | 95 | 92 |
|  |  |  |  |  |  |  |  |
| Normal control group (mean±SD) (beats) | 250±0 | 300±0 | 395.7±20.2 | 489.4±39.1 | 578.6±65.9 | 663.6±96.1 | 740.2±128.6 |
| Mean achievement rate (%) | 100 | 100 | 99 | 98 | 96 | 95 | 93 |
|  |  |  |  |  |  |  |  |

| Table S2. LP parameter changes according to an increment of averaging time (night-time) |
| --- |

|  |  |  |  |  |  |  |  |  |  |
| --- | --- | --- | --- | --- | --- | --- | --- | --- | --- |
| **fQRS** |  |  |  |  |  |  |  |  |  |
| Averaging beats |  | 250 | 300 | 400 | 500 | 600 | 700 | 800 | p |
| MI-VT group (ms) | 25 percentile | 105.5 | 104.0 | 105.5 | 105.5 | 105.5 | 105.5 | 106.0 |  |
| n=30 (ms) | 50 percentile | 112.0 | 112.0 | 113.0 | 113.0 | 114.0 | 115.0 | 117.0 | <0.001 |
|  | 75 percentile | 138.0 | 138.0 | 138.0 | 138.0 | 139.5 | 139.5 | 140.0 |  |
|  |  |  |  |  |  |  |  |  |  |
| MI non-VT group | 25 percentile | 93.0 | 93.8 | 93.8 | 93.8 | 93.8 | 94.0 | 94.0 |  |
| (ms) | 50 percentile | 102.0 | 102.0 | 103.0 | 103.0 | 102.5 | 102.5 | 102.5 | 0.319 |
| n=74 | 75 percentile | 112.5 | 112.5 | 113.3 | 113.3 | 114.0 | 113.3 | 113.3 |  |
|  |  |  |  |  |  |  |  |  |  |
| Normal control | mean | 91.4 | 91.2 | 91.1 | 91.1 | 91.2 | 91.2 | 91.2 | 0.248 |
| group (ms), n=86 | SD | 7.5 | 7.5 | 7.6 | 7.6 | 7.4 | 7.5 | 7.5 |  |
|  |  |  |  |  |  |  |  |  |  |
| **LAS40** |  |  |  |  |  |  |  |  |  |
| Averaging beats |  | 250 | 300 | 400 | 500 | 600 | 700 | 800 | p |
| MI-VT group (ms) | 25 percentile | 26.0 | 26.5 | 27.5 | 27.5 | 28.0 | 28.0 | 28.0 |  |
| n=30 (ms) | 50 percentile | 35.0 | 34.0 | 36.0 | 36.0 | 37.0 | 38.0 | 38.0 | <0.001 |
|  | 75 percentile | 47.5 | 47.5 | 47.5 | 47.5 | 48.0 | 48.0 | 48.0 |  |
|  |  |  |  |  |  |  |  |  |  |
| MI non-VT group | 25 percentile | 23.0 | 23.0 | 24.0 | 24.0 | 24.0 | 24.0 | 23.8 |  |
| (ms) | 50 percentile | 31.0 | 30.5 | 31.0 | 31.5 | 31.5 | 31.5 | 31.5 | 0.321 |
| n=74 | 75 percentile | 40.3 | 40.3 | 39.8 | 40.3 | 40.0 | 40.0 | 40.3 |  |
|  |  |  |  |  |  |  |  |  |  |
| Normal control | mean | 29.7 | 29.7 | 29.6 | 29.7 | 29.6 | 29.6 | 29.6 | 0.826 |
| group (ms), n=86 | SD | 7.2 | 7.2 | 7.4 | 7.3 | 7.4 | 7.3 | 7.3 |  |
|  |  |  |  |  |  |  |  |  |  |
| **RMS40** |  |  |  |  |  |  |  |  |  |
| Averaging beats |  | 250 | 300 | 400 | 500 | 600 | 700 | 800 | p |
| MI-VT group (μV) | 25 percentile | 12.0 | 12.0 | 12.0 | 12.0 | 11.0 | 12.0 | 12.0 |  |
| n=30 (ms) | 50 percentile | 24.0 | 25.0 | 24.0 | 24.0 | 24.0 | 22.0 | 22.0 | <0.001 |
|  | 75 percentile | 51.5 | 49.5 | 48.5 | 48.5 | 44.0 | 43.5 | 43.0 |  |
|  |  |  |  |  |  |  |  |  |  |
| MI non-VT group | 25 percentile | 18.8 | 18.8 | 17.5 | 17.5 | 17.5 | 17.5 | 17.5 |  |
| (µV) | 50 percentile | 29.5 | 29.5 | 29.0 | 29.0 | 29.0 | 29.0 | 29.0 | 0.411 |
| n=74 | 75 percentile | 48.0 | 46.8 | 45.8 | 45.3 | 45.8 | 45.8 | 47.3 |  |
|  |  |  |  |  |  |  |  |  |  |
| Normal control | 25 percentile | 28.0 | 28.0 | 27.8 | 27.8 | 27.8 | 27.8 | 27.8 |  |
| group (µV) | 50 percentile | 45.0 | 45.0 | 45.0 | 45.0 | 46.0 | 46.0 | 46.0 | 0.716 |
| n=86 | 75 percentile | 64.0 | 67.3 | 66.3 | 66.3 | 66.3 | 65.5 | 65.5 |  |
| fQRS=filtered QRS duration, LAS40=duration of low-amplitude signals <40 μV in the terminal filtered QRS complex, RMS40=root mean square voltage of the terminal 40 ms in the filtered QRS complex | | | | | | | | | |

| Table S3. LP parameter changes according to an increment of averaging time (daytime) | | | | | | | | | |
| --- | --- | --- | --- | --- | --- | --- | --- | --- | --- |
|  | | | | | | | | | |
| **fQRS** | | | | | | | | |  |
| Averaging beats |  | 250 | 300 | 400 | 500 | 600 | 700 | 800 | p |
| MI-VT group (ms) | 25 percentile | 104.0 | 104.0 | 104.0 | 105.0 | 105.5 | 105.5 | 105.5 |  |
| n=30 | 50 percentile | 116.0 | 117.0 | 117.0 | 117.0 | 117.0 | 117.0 | 117.0 | <0.001 |
|  | 75 percentile | 136.5 | 136.5 | 137.5 | 137.5 | 137.5 | 137.5 | 137.0 |  |
|  |  |  |  |  |  |  |  |  |  |
| MI non-VT | 25 percentile | 89.8 | 89.8 | 90.0 | 90.0 | 90.0 | 90.0 | 90.0 |  |
| group (ms) | 50 percentile | 98.0 | 98.0 | 98.0 | 98.5 | 98.5 | 98.5 | 98.5 | 0.180 |
| n=74 | 75 percentile | 110.3 | 111.0 | 111.0 | 111.0 | 111.0 | 111.0 | 111.0 |  |
|  |  |  |  |  |  |  |  |  |  |
| Normal control | mean | 86.3 | 85.9 | 85.8 | 86.0 | 86.1 | 86.2 | 86.1 | 0.110 |
| group (ms), n=86 | SD | 8.1 | 8.1 | 7.8 | 7.9 | 7.8 | 7.8 | 7.8 |  |
|  | | | | | | | | |  |
| **LAS40** | | | | | | | | |  |
| Averaging beats |  | 250 | 300 | 400 | 500 | 600 | 700 | 800 |  |
| MI-VT group (ms) | 25 percentile | 26.5 | 26.5 | 26.5 | 27.0 | 27.0 | 27.0 | 27.0 |  |
| n=30 | 50 percentile | 38.0 | 38.0 | 39.0 | 39.0 | 40.0 | 39.0 | 39.0 | <0.001 |
|  | 75 percentile | 48.5 | 48.5 | 49.5 | 48.0 | 48.5 | 48.0 | 48.0 |  |
|  |  |  |  |  |  |  |  |  |  |
| MI non-VT group | 25 percentile | 22.8 | 22.8 | 22.8 | 23.0 | 23.0 | 23.0 | 23.0 |  |
| (ms) | 50 percentile | 29.0 | 29.0 | 29.0 | 29.0 | 29.0 | 29.0 | 29.0 | 0.142 |
| n=74 | 75 percentile | 34.0 | 34.0 | 34.0 | 34.3 | 34.3 | 34.3 | 34.3 |  |
|  |  |  |  |  |  |  |  |  |  |
| Normal control | mean | 26.8 | 26.5 | 26.8 | 26.8 | 26.8 | 26.9 | 26.8 | 0.274 |
| group (ms), n=86 | SD | 7.3 | 7.3 | 7.2 | 7.1 | 7.0 | 7.1 | 7.1 |  |
|  |  |  |  |  |  |  |  |  |  |
| **RMS40** | | | | | | | | |  |
| Averaging beats |  | 250 | 300 | 400 | 500 | 600 | 700 | 800 |  |
| MI-VT group (μV) | 25 percentile | 11.0 | 11.0 | 11.0 | 11.0 | 12.0 | 12.0 | 12.0 |  |
| n=30 | 50 percentile | 24.0 | 24.0 | 19.0 | 20.0 | 20.0 | 19.0 | 18.0 | <0.001 |
|  | 75 percentile | 55.0 | 51.0 | 51.0 | 51.0 | 47.5 | 47.5 | 46.0 |  |
|  |  |  |  |  |  |  |  |  |  |
| MI non-VT group | 25 percentile | 22.0 | 21.8 | 21.0 | 21.0 | 21.0 | 21.0 | 21.8 |  |
| (µV) | 50 percentile | 36.5 | 36.0 | 37.0 | 36.5 | 36.5 | 37.0 | 36.0 | 0.627 |
| n=74 | 75 percentile | 52.0 | 49.0 | 49.5 | 49.0 | 50.3 | 50.3 | 51.0 |  |
|  |  |  |  |  |  |  |  |  |  |
| Normal control | 25 percentile | 33.3 | 27.6 | 33.0 | 33.0 | 33.8 | 33.8 | 33.8 |  |
| group (µV) | 50 percentile | 58.0 | 58.0 | 58.0 | 58.0 | 58.0 | 57.0 | 57.0 | 0.089 |
| n=86 | 75 percentile | 83.0 | 82.5 | 82.5 | 83.5 | 84.3 | 83.3 | 83.3 |  |
| Abbreviation as in Supplementary Table 2-1 | | | | | | | | | |

| Table S4. Noise level changes according to increment of averaging times (night-time) |
| --- |

|  |  |  |  |  |  |  |  |  |  |
| --- | --- | --- | --- | --- | --- | --- | --- | --- | --- |
| Averaging beats |  | 250 | 300 | 400 | 500 | 600 | 700 | 800 | p |
| MI-VT group (µV) (µV) | 25 Percentile | 0.23 | 0.21 | 0.19 | 0.18 | 0.17 | 0.16 | 0.15 |  |
| (µV) | 50 Percentile | 0.26 | 0.25 | 0.21 | 0.22 | 0.21 | 0.20 | 0.20 | <0.001 |
|  | 75 Percentile | 0.36 | 0.33 | 0.28 | 0.26 | 0.25 | 0.25 | 0.23 |  |
|  |  |  |  |  |  |  |  |  |  |
| Averaging beats |  | 250 | 300 | 400 | 500 | 600 | 700 | 800 | p |
| MI non-VT group | 25 Percentile | 0.24 | 0.22 | 0.19 | 0.19 | 0.17 | 0.16 | 0.15 |  |
| (µV) | 50 Percentile | 0.29 | 0.27 | 0.24 | 0.24 | 0.21 | 0.22 | 0.21 | <0.001 |
|  | 75 Percentile | 0.39 | 0.38 | 0.34 | 0.31 | 0.28 | 0.27 | 0.27 |  |
|  |  |  |  |  |  |  |  |  |  |
| Averaging beats |  | 250 | 300 | 400 | 500 | 600 | 700 | 800 | p |
| Normal control | 25 Percentile | 0.22 | 0.21 | 0.18 | 0.17 | 0.15 | 0.14 | 0.13 |  |
| group (µV) | 50 Percentile | 0.26 | 0.24 | 0.21 | 0.20 | 0.19 | 0.17 | 0.17 | <0.001 |
|  | 75 Percentile | 0.32 | 0.33 | 0.29 | 0.27 | 0.24 | 0.25 | 0.24 |  |

| Table S5. Noise level changes according to increment of averaging times (daytime) | | | | | | | | | |  |
| --- | --- | --- | --- | --- | --- | --- | --- | --- | --- | --- |
|  | | | | | | | | |  |  |
| Averaging beats |  | 250 | 300 | 400 | 500 | 600 | 700 | 800 | p | |
| MI-VT group (µV) | 25 Percentile | 0.31 | 0.33 | 0.28 | 0.25 | 0.21 | 0.21 | 0.20 |  | |
| (µV) | 50 Percentile | 0.43 | 0.41 | 0.35 | 0.33 | 0.32 | 0.28 | 0.29 | <0.001 | |
|  | 75 Percentile | 0.67 | 0.59 | 0.55 | 0.47 | 0.44 | 0.42 | 0.40 |  | |
|  |  |  |  |  |  |  |  |  |  | |
| Averaging beats |  | 250 | 300 | 400 | 500 | 600 | 700 | 800 | p | |
| MI non-VT group | 25 Percentile | 0.33 | 0.33 | 0.27 | 0.28 | 0.26 | 0.25 | 0.25 |  | |
| (µV) | 50 Percentile | 0.51 | 0.52 | 0.47 | 0.42 | 0.40 | 0.39 | 0.38 | <0.001 | |
|  | 75 Percentile | 0.75 | 0.69 | 0.66 | 0.61 | 0.61 | 0.61 | 0.59 |  | |
|  |  |  |  |  |  |  |  |  |  | |
| Averaging beats |  | 250 | 300 | 400 | 500 | 600 | 700 | 800 | p | |
| Normal control | 25 Percentile | 0.36 | 0.36 | 0.31 | 0.28 | 0.32 | 0.30 | 0.29 |  | |
| group (µV) | 50 Percentile | 0.55 | 0.53 | 0.52 | 0.50 | 0.49 | 0.46 | 0.45 | <0.001 | |
|  | 75 Percentile | 0.74 | 0.71 | 0.68 | 0.68 | 0.68 | 0.63 | 0.62 |  | |

| Table S6. Patient demographics between LP change (+) vs LP change (-) in the MI-VT group | | | | | |  |  |
| --- | --- | --- | --- | --- | --- | --- | --- |
| Demographics | LP determinate change (+)  group (n=6) | | LP  determinate change (-)  group (n=24) | | p-value |  |  |
|  |  |  |  |  |  |  |  |
| Age (years) | 70.5 [66.0, 77.8] | | 68.5 [61.3, 76.0] | | 0.713 |  |  |
| Sex: male, n (%) | 6 (100) | | 23 (96) | | 0.555 |  |  |
| Hypertension, n (%) | 6 (100) | | 24 (100) | | 1 |  |  |
| Dyslipidemia, n (%) | 3 (50) | | 13 (54) | | 0.149 |  |  |
| Diabetes mellitus, n (%) | 4 (67) | | 17 (71) | | 0.193 |  |  |
| Coronary culprit lesion |  |  |  |  |  |  |  |
| RCA | 2 (33) | | 12 (50) | | — |  |  |
| LAD | 4 (67) | | 19 (79) | | 0.092 |  |  |
| Cx | 1 (17) | | 8 (33) | | — |  |  |
| Single vessel disease | 4 (67) | | 12 (50) | | 0.657 |  |  |
| Two vessel disease | 1 (16) | | 7 (29) | | — |  |  |
| Three vessel disease | 1 (16) | | 5 (21) | | — |  |  |
| Left main tract | 0 (0) | | 0 (0) | | — |  |  |
| CABG | 1 (16) | | 4 (33) | | — |  |  |
| Echocardiographic data |  |  |  |  |  |  |  |
| LVEF (%) | 40.2 [32.6, 56.7] | | 44.5 [31.6, 63.1] | | 0.648 |  |  |
| LVDd (mm) | 57.0 [50.9, 56.7] | | 58.5 [48.7, 66.5] | | 0.978 |  |  |
|  |  | |  | |  |  |  |
| Creatine kinase (U/L) | 599 [296, 5712] | | 632 [254, 6513] | | 0.344 |  |  |
| Troponin I (ng/mL) | 14.2 [1.7, 23.5] | | 15.8 [1.7, 28.1] | | 0.421 |  |  |
|  |  | |  | |  |  |  |
| NSVT | 2 (33) | | 10 (42) | | — |  |  |
| Paroxysmal atrial fibrillation | 1 (16) | | 4 (33) | | — |  |  |
|  |  | |  | |  |  |  |
| Renal function |  | |  | |  |  |  |
| Estimate GFR, mL/min per 1.73 m^2^ | 46.7 [33.5, 76.1] | | 47.3 [35.3, 70.9] | | 0.924 |  |  |
|  |  |  |  |  |  |  |  |
| NYHA functional class |  |  |  |  |  |  |  |
| NYHA I (%) | 2 (33) | | 12 (86) | | — |  |  |
| NYHA II (%) | 2 (33) | | 7 (29) | | 0.41 |  |  |
| NYHA III (%) | 2 (33) | | 3 (13) | | — |  |  |
| NYHA IV (%) | 0 (0) | | 2 (8) | | — |  |  |
| Therapy |  |  |  |  |  |  |  |
| β-Blocker (%) | 5 (83) | | 21 (88) | | 0.565 |  |  |
| RAS-inhibitor (%) | 5 (84) | | 20 (83) | | 0.449 |  |  |
| CCB (%) | 3 (50) | | 15 (63) | | 0.091 |  |  |
| Diuretic (%) | 4 (67) | | 15 (63) | | 0.091 |  |  |
| Amiodarone (%) | 4 (67) | | 6 (25) | | 0.414 |  |  |
| Mexiletine (%) | 0 (0) | | 1 (4) | | — |  |  |
| Pilsicainide (%) | 0 (0) | | 0 | | — |  |  |
| Data are presented as n (%) or mean SD; CABG=coronary artery bypass grafting, CAG=coronary artery angiography, CCB=calcium channel blockers, CX=circumflex branch, LAD=left anterior descending, LVEF=left ventricular ejection fraction, LVDd=left ventricular dimension diameter, NYHA=New York Heart Association, NSVT=non-sustained ventricular tachycardia, RAS=renin–angiotensin system, RCA=right coronary artery. | | | | | |  |  |
|  |  |  |  |  |  |  |  |
|  |  |  |  |  |  |  |  |
|  |  |  |  |  |  |  |  |
